# Associations between Recreational and Commuter Cycling, Changes in Cycling, and Type 2 Diabetes Risk: A Cohort Study of Danish Men and Women

**DOI:** 10.1371/journal.pmed.1002076

**Published:** 2016-07-12

**Authors:** Martin G. Rasmussen, Anders Grøntved, Kim Blond, Kim Overvad, Anne Tjønneland, Majken K. Jensen, Lars Østergaard

**Affiliations:** 1 Research Unit for Exercise Epidemiology, Department of Sports Science and Clinical Biomechanics, University of Southern Denmark, Odense, Denmark; 2 Department of Cardiology, Aalborg University Hospital, Aalborg, Denmark; 3 Department of Public Health, Section for Epidemiology, Aarhus University, Aarhus, Denmark; 4 Danish Cancer Society Research Center, Copenhagen, Denmark; 5 Department of Nutrition, Harvard T.H. Chan School of Public Health, Boston, Massachusetts, United States of America; 6 Department of Medicine, Channing Division of Network Medicine, Harvard Medical School, Brigham and Women's Hospital, Boston, Massachusetts, United States of America; University of Cambridge, UNITED KINGDOM

## Abstract

**Background:**

Cycling is a recreational activity and mode of commuting with substantial potential to improve public health in many countries around the world. The aim of this study was to examine prospective associations between recreational and commuter cycling, changes in cycling habits, and risk of type 2 diabetes (T2D) in Danish adults from the Diet, Cancer and Health cohort study.

**Methods and Findings:**

At baseline from 1993 to 1997, 24,623 men and 27,890 women from Denmark, 50–65 y of age and free of T2D and other chronic diseases, underwent a number of assessments, including completing a lifestyle questionnaire also addressing cycling habits. Approximately 5 y later, at a second examination, participants completed a new, updated lifestyle questionnaire. Cox regression was used to estimate hazard ratios (HRs) of incident T2D registered in the Danish National Diabetes Registry, according to recreational and commuter cycling and changes in cycling habits, with adjustment for a priori known T2D risk factors. During 743,245.4 person-years of follow-up (mean follow-up 14.2 y), 6,779 incident cases of T2D were documented. Multivariable adjusted HRs (95% confidence interval [CI]) were 1, 0.87 (0.82, 0.93), 0.83 (0.77, 0.89), 0.80 (0.74, 0.86) and 0.80 (0.74, 0.87) (*p* for trend = <0.001) for 0, 1–60, 61–150, 151–300, and >300 min/wk of total cycling (recreational and commuter cycling), respectively. In analysis of seasonal cycling, multivariable adjusted HRs (95% CI) were 1, 0.88 (0.83, 0.94), and 0.80 (0.76, 0.85) for non-cyclists, seasonal cyclists (those cycling only in summer or winter), and those cycling during both summer and winter, respectively. How changes in total cycling from baseline to the second examination affected risk was also investigated, and multivariable adjusted HRs (95% CI) were 1, 0.88 (0.78, 1.01), 0.80 (0.69, 0.91), and 0.71 (0.65, 0.77) for non-cyclists and for those who ceased, initiated, or continued cycling between baseline and the second examination, respectively. Lastly, in the analysis of commuter cycling, multivariable HRs (95% CI) were 1, 0.72 (0.60, 0.87), 0.83 (0.69, 1.00), and 0.70 (0.57, 0.85) (*p* for trend = <0.001) for cycling 0, 1–60, 61–150, and >150 min/wk to work, respectively. The main limitation of the current study is the use of self-reported physical activity.

**Conclusions:**

Commuter and recreational cycling was consistently associated with lower risk of T2D in Danish adults. Our results also provide evidence that late-in-life initiation of or continued engagement in cycling lowers risk of T2D.

## Abstract 2

Martin Rasmussen and colleagues show a reduced incidence of type 2 diabetes for cyclists, even for those who start later in life.

## Author Summary

**Why Was this Study Done?:**

- Habitual cycling in the general population may have the potential to promote public health by reducing the risk of chronic diseases, such as type 2 diabetes (T2D).
- A number of previous studies have investigated the relationship between active transport, combining cycling and walking, and T2D; however, previous research investigating the relationship between cycling for recreation or transport and risk of T2D is sparse.
- Our study was done to investigate whether cycling specifically is valuable in the prevention of T2D among a Danish population with widespread engagement in regular cycling.

**What Did the Researchers Do and Find?:**

- Based on information from a large population-based study, we investigated if the risk of T2D was related to habitual cycling when personal- and lifestyle-related risk factors were taken into account.
- We found that habitual cycling was related to a lower risk of T2D than no cycling, and that a longer duration of weekly cycling seemed to be preferable to less weekly cycling.
- We also found that those who took up habitual cycling in middle to old age had a 20% lower risk of T2D compared to those who remained non-cyclists.

**What Do These Findings Mean?:**

- It seems beneficial to encourage adults of middle and old age to engage in commuter and recreational cycling to prevent the development of T2D in late adulthood.
- When combining our findings with previous studies that have shown it is possible to increase cycling through promotional activities and infrastructural changes, it may be suggested that national and local governments prioritize resources to promote cycling.

## Introduction

Cycling for transportation and recreation has been emphasized for its great potential for improving public health [1]. As part of an overall global action plan to prevent and control noncommunicable disease, the World Health Organization has called for a reduction in the prevalence of physical inactivity. The promotion of active transport, i.e., commuter walking or cycling, is a central component of the policy options defined to achieve this goal [2]. Because cycling may be conveniently incorporated into daily life chores such as commuting to school, work, or grocery shopping, it may be a mode of physical activity that appeals to a large part of the population. Additionally, cycling is an environmentally sustainable mode of transport compared to motorized transportation [3]. Although habitual cycling is likely to be health- and fitness-enhancing physical activity [4], its importance for chronic disease prevention in populations has been scarcely investigated. Habitual cycling has, in prospective cohort studies of adults, been associated with a lower incidence of fatal and non-fatal cardiovascular disease [5], less weight gain [6], and lower all-cause mortality [7]. Cycling may be particularly valuable for type 2 diabetes (T2D) prevention, since a large body of evidence from observational and experimental studies shows that regular engagement in physical activity and lifestyle intervention incorporating physical activity substantially decreases T2D risk [8].

The benefits of recreational and commuter cycling for the prevention of T2D are still unclear; cycling has been associated with a lower risk of diabetes cross-sectionally [9–12], and two prospective studies found no lower risk of incident T2D when comparing cyclists with non-cyclists who were free of chronic disease at recruitment [13,14]. It remains to be examined in greater detail whether cycling is valuable in the prevention of T2D. The purpose of this study was to investigate the prospective association of recreational and commuter cycling with T2D risk among generally healthy Danish adults in the Diet, Cancer and Health cohort study. We also investigated how seasonal cycling and changes in cycling habits were related to incidence of T2D. We undertook these investigations in a Danish adult population living in cities where cycling infrastructure is well developed and cycling is common across socio-demographic groups [3].

## Methods

### Ethics

The Diet, Cancer and Health study was conducted in accordance with the Helsinki Declaration. It was approved by the Scientific Ethical Committee of Copenhagen (no. H-KF-01-345/93), and the study protocol for the current study was approved by the Danish Data Protection Agency (no. 2015-57-0008). At the study clinic, informed written consent to collect data on health outcomes in medical registries in the years that followed was gathered from all study participants [15].

### Participants

Between 1993 and 1997, 80,996 men and 79,729 women were invited to participate in the Diet, Cancer and Health study. Inhabitants of Aarhus, Copenhagen, and surrounding cities were invited if between 50 and 64 years of age, born in Denmark, and without a diagnosis of cancer registered in the Danish Cancer Registry. Nineteen percent of the Danish population within the eligible age group were invited. Age of eligibility was chosen based on the prospect that new cases of cancer and other chronic diseases would occur within a relatively short time frame. Eligible persons were identified through the Civil Registration System, and 27,178 men and 29,875 women agreed to participate in baseline examinations [15], which took place between November 24th, 1993, and May 28th, 1997. In analysis of total cycling (combined recreational and commuter cycling) and seasonal cycling, which was reported at baseline, the following disease cases were excluded: diabetes according to the National Diabetes Registry (NDR), the National Patient Registry, or self-report (*n* = 1,395); acute myocardial infarction (*n* = 913); stroke (*n* = 597); and cancer (*n* = 569). This corresponded to exclusion of 3,268 participants registered with one or more chronic diseases, leaving 53,785 eligible participants. After excluding missing data in covariates and exposure variables, 52,513 participants were included in the analyses.

**Fig 1:**
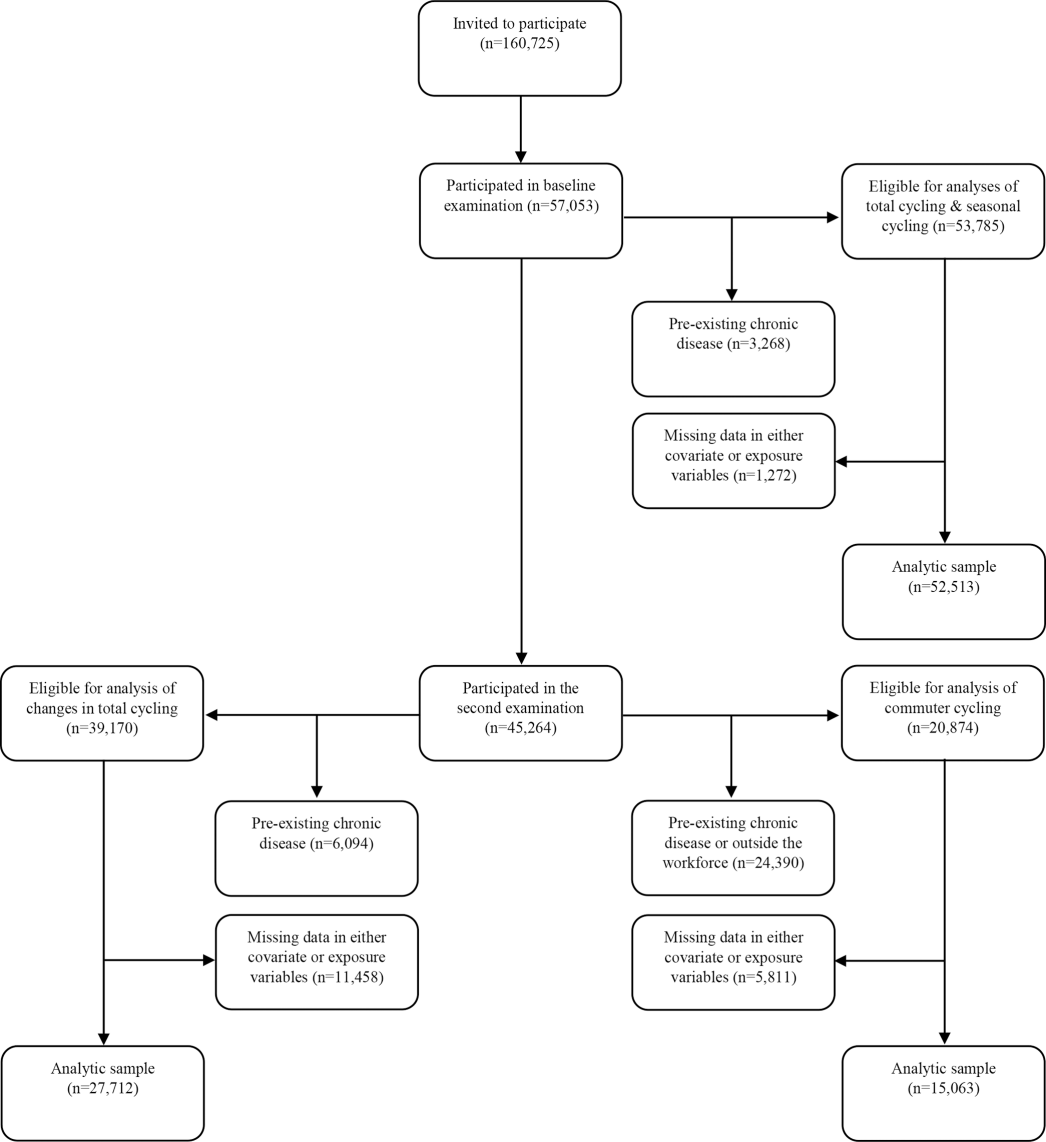
Flow chart of participants from invitation to analyses. The following chronic diseases were excluded: diabetes (any diabetes diagnosis), acute myocardial infarction, stroke, and cancer. “Outside the workforce” refers to participants who are either unemployed or retired. Because participants must necessarily participate in the second examination to be included in the analysis of changes in total cycling, flow of participants for this analysis is shown following those who participate in the second examination.

Approximately 5 y later (mean 5.4 y), between 1999 and 2003, participants still alive and residing in Denmark were invited for a second examination, and 45,264, or 79.3%, of the original cohort participated between September 18th, 1999, and March 20th, 2003. In analysis of changes in total cycling from baseline to the second examination, new disease cases between the two examinations were excluded, in addition to those listed above: diabetes according to the NDR, the National Patient Registry, or self-report (*n* = 1,485); acute myocardial infarction (*n* = 463); stroke (*n* = 368); and cancer (*n* = 2,206). This corresponded to exclusion of 4,328 participants registered with one or more chronic diseases. When also excluding those registered with one or more chronic diseases before baseline, a total of 6,094 participants were excluded, leaving 39,170 eligible participants. When those with missing data in covariates and exposure variables were excluded, 27,712 participants were included in the analysis. For analysis of commuter cycling, which was reported at the second examination, unemployed individuals (*n* = 1,375) and those who had retired (*n* = 20,738) were also excluded, leaving 20,874 eligible participants. After excluding missing data in covariates and exposure variables, 15,063 participants were included in the analysis (see Fig 1).

### Data Collection

At baseline, a validated semi-quantitative food frequency questionnaire, which was developed to be compatible with the Danish diet [16–19], was sent by mail and filled out before a visit to a study clinic. At the clinic, an additional questionnaire was completed, containing lifestyle questions in general, addressing habits pertaining to, e.g., physical activity, smoking, and alcohol. Furthermore, a lab technician measured anthropometrics, including weight, height, and waist circumference, and biological material was collected [15].

At the second examination, a similar dietary survey, which also included questions on foods that since baseline had been introduced to the Danish diet, was sent by mail and completed in the participants’ home. An updated lifestyle questionnaire was also sent to the participants, e.g., introducing questions on social network and self-rated health from the Medical Outcomes Study 36-item short-form survey. Enquiry of weight according to self-report was included in the lifestyle questionnaire. A tape measure was sent also to self-assess waist circumference [15].

### Assessment of Physical Activity

In the lifestyle questionnaires, physical activity was enquired for both summer and winter, except for physical activity at work and for stair climbing. Participants were asked to report total hours per week of physical activity, although at the second examination, frequency of the activity was specified, as well as duration per time the activity was performed. This information was used to calculate total hours per week for each activity. At baseline, the following physical activities were reported: physical activity at work, walking, total cycling, housework, do-it-yourself work, gardening, sports participation, and stair climbing. The same physical activities, except for stair climbing, were reported at the second examination, although sports participation was divided into light, moderate, and vigorous sport. Importantly, at baseline, cycling was reported in total, i.e., recreational cycling and commuter cycling combined, whereas at the second examination, recreational cycling and commuter cycling were reported separately.

The physical activity questions in the baseline questionnaire have previously been validated in populations similar to the Diet, Cancer and Health cohort against heart rate monitors to assess energetic expenditure [20], accelerometers [21], and heart rate and movement censors [22]. The studies showed that the questions were valid in terms of ranking groups of participants in large epidemiological studies according to overall physical activity levels. The results also revealed fair reliability, with a significant weighed kappa statistic of 0.6 [20] and Spearman correlation coefficients of 0.58–0.73. Fair reliability was also shown for cycling specifically (Spearman correlation coefficient = 0.65) [21]. An assessment of the reliability and validity of the questionnaire used at second examination has shown moderate-to-high reliability, with an intra-class correlation coefficient of 0.76 for physical activity energy expenditure and good validity for ranking individuals according to their overall physical activity energy expenditure [23].

Physical activity variables were aggregated to average weekly minutes of physical activity from winter and summer values. Furthermore, composite leisure time physical activity variables were created in metabolic equivalent (MET) hours per week based on the following: 3 METs for walking or housework, 4 METs for gardening, 4.5 METs for do-it-yourself work, 6 METs for cycling and for general sports, and 4.5 METs, 8.5 METs, and 10 METs for light, moderate, and vigorous sport participation, respectively. One MET is considered equivalent to the resting metabolic rate, and MET values express intensity levels as multiples of the resting metabolic rate [24]. Leisure time physical activity variables, including leisure time physical activity other than relevant cycling, were also generated to adjust for these in the analyses.

Total cycling was categorized in 0, 1–60, 61–150, 151–300, and >300 min/wk. When achieving >150 min/wk, one successfully met international recommendations for aerobic physical activity [25]. Subjects were also categorized according to seasonal cycling as non-cyclist, cyclists only summer or winter, or cyclist summer and winter. Because few participants reported cycling only in winter, it was not possible to create separate categories for winter cycling only and summer cycling only. Subjects were defined as cyclists if values were above 0 min/wk.

To define changes in total cycling from baseline to the second examination, we first computed total cycling at the second examination by adding recreational and commuter cycling. Participants were then grouped according to those who did no cycling, ceased to cycle, initiated cycling, or continued cycling in the period between baseline and the second examination. The cumulative average of total cycling from the two measurement points was also computed to represent level of long-term exposure.

Commuter cycling was categorized similarly as total cycling, although because there were few incident T2D cases in the two highest categories, these were merged to >150 min/wk.

### Follow-Up and Identification of T2D Cases

All participants were followed through national disease registries through their unique person identification number in the Danish Civil Registration System [26]. Cases of diabetes were identified based on registration in the Danish NDR. Established in 2006, the NDR identifies cases based on data from other Danish medical registries. If one of six specific criteria in one of three registries is met, this person is included. These criteria are: a diagnosis of diabetes according to the National Patient Registry; registration of diabetic chiropody, i.e., treatment of feet when also registered as a diabetic patient, in the National Health Service Register; five annual blood glucose measurements or two blood glucose measurements per year for five consecutive years, according to the National Health Service Register; and, lastly, purchase of anti-diabetic drugs or prescribed insulin, registered in the Danish National Prescription Registry. The registry also contains the date of inclusion [27]. This method of identifying cases of diabetes has shown to have a sensitivity of 91% and a positive predictive value of 89% [28]. Personal data, such as vital status from the Danish Civil Registration System, is also included in the NDR. Although the NDR does not include data on type of diabetes diagnosis [27], incident cases of diabetes in this study population are, however, interpreted as T2D, as adult-onset type 1 diabetes has been found to be low proportionally to adult-onset T2D [29,30].

### Statistical Methods

Due to asymmetric distributions, descriptive data for continuous variables were computed as medians with interquartile ranges. Categorized data were computed as proportions.

If participants had only answered for one season in physical activity variables, the value for the unanswered season was imputed based on the means for summer and winter for participants of the same sex and age (six strata) who had answered the question for both seasons. For participants included in the analyses of total cycling and seasonal cycling, <0.2% had only answered for one season in the physical activity variables; among participants in the analysis of changes in total cycling, this ranged between 0.6% and 5.7%. Lastly, in analysis of commuter cycling, the proportion who had only answered for one season ranged between 0.5% and 5.4%. The multivariable analyses performed without imputation of physical activity variables, however, showed almost identical results as the analyses with imputation, with no differences in direction of associations or statistical significance.

A few individuals reported unrealistically high values of cycling. Values above 18 hrs/wk and 21 hrs/wk were considered unrealistic for commuter cycling and recreational cycling, respectively. Total cycling considered realistic was 28 hrs/wk. Variables were truncated at these maximum values if the change influenced total and within-category means.

Hazard ratios (HRs) with 95% confidence intervals (CIs) of developing T2D according to cycling habits were estimated using Cox proportional hazard regression. Proportionality of hazards was assessed in adjusted log-log plots, and no systematic deviation from proportionality was found. The underlying time scale was age, and the event was registration in the NDR. Registrations in NDR prior to January 1st, 1995, have previously been found to be imprecise [31], and, thus, cases preceding this date (*n* = 19) were excluded. In analyses of total cycling and seasonal cycling, participants were considered at risk from age at clinic visit at baseline. In analysis of changes in total cycling and in analysis of commuter cycling, participants were considered at risk from age at the second examination. Right truncation was at age of the following: event, death, emigration, inactivity, or change in the Civil Registration System, or end of follow-up (December 31st, 2011), whichever came first.

Test for trend was computed in analyses of total cycling and commuter cycling by assigning participants with the median of their respective exposure category and treating this as continuous in the model.

Confounders included in the Cox models were selected based on knowledge a priori. For each analysis, an age-adjusted and a multivariable-adjusted model was computed. In analysis of total cycling and seasonal cycling, the following variables were adjusted for: age at baseline (years), sex (male/female), years of basic school (<7/8–10/>10), years of higher education (0/1–2/3–4/>4), alcohol intake (grams/day), dietary energy intake (kJ/day), vegetable intake (grams/day), fruit intake (grams/day), potato consumption (grams/day), wholegrain cereal consumption (grams/day), refined cereal consumption (grams/day), glycaemic load of all carbohydrates (units/day), coffee consumption (grams/day), polyunsaturated:saturated fat ratio, physical activity at work (no work/sedentary/standing/manual work/heavy manual work), smoking status (never/former/<15 g per day/15–25 g per day/>25 g per day), and leisure-time physical activity other than total cycling (MET*hours per week). In the analysis of changes in total cycling from baseline to the second examination, the following covariates were included in the model: age at the second examination (years), sex (male/female), years of basic school (<7/8–10/>10), years of higher education (0/1–2/3–4/>4), alcohol intake (grams/day), dietary energy intake (kJ/day), coffee consumption (grams/day), polyunsaturated:saturated fat ratio, physical activity at work (no work/sedentary/standing/manual work/heavy manual work), smoking status (never/former/<15 g per day/15–25 g per day/>25 g per day), paternal diabetes (yes/no/do not know), maternal diabetes (yes/no/do not know), and leisure-time physical activity other than total cycling (MET*hours per week). Other than sex, years of basic school, years of higher education, and physical activity at work, these were all based on data from the second examination. In the analysis of commuter cycling, the adjustment was similar, although leisure-time physical activity other than commuter cycling was included instead of leisure-time physical activity other than total cycling. Dietary variables on a continuous scale were categorized into quintiles, although coffee consumption was categorized into quartiles. Leisure-time physical activity variables were also categorized into quintiles. Age was treated as continuous in the model, assuming a linear relationship between age and risk of T2D. In all analyses, adjustment for waist circumference was included in addition to the above in separate models, to investigate the role of waist circumference as a potential mediating factor in the causal link between cycling and T2D. Analyses with adjustment for body mass index (BMI) were also performed for the same purpose. Importantly, BMI and waist circumference were measured objectively at baseline and subjectively at the second examination, where BMI at the second examination was calculated based on height at baseline and self-reported weight at the second examination.

We also statistically evaluated whether there was an interaction between sex and cycling on risk of T2D in the analyses. This was done by comparing models with an interaction term and main effects with models containing main effects only, using the likelihood-ratio test. No interaction of sex was found in any analyses.

All analyses were conducted using STATA IC V.14 (STATA Corp, College Station, Texas, USA) with α = 0.05.

## Results

The current study included 743,245.4 person-years of follow-up (mean follow-up 14.2 years) from 52,513 participants, in which 6,779 incident cases of T2D were documented. This corresponded to an incidence rate (95% CI) of 9.1 (8.9,9.3) cases per 1,000 years of follow-up.

At baseline, cyclists differed from non-cyclists on most characteristics (Table 1). With higher weekly time spent cycling, median waist circumference was lower. In relation to physical activity behaviours, leisure-time physical activity other than cycling tended to be higher with cycling. Also, for physical activity at work, the proportion reporting sedentary physical activity at work tended to be lower and the proportion reporting manual work higher. With higher reported cycling, daily alcohol intake tended to be lower. In several dietary variables, i.e., energy intake, vegetable and fruit consumption, wholegrain cereal consumption, and glycaemic load of all carbohydrates, these were higher with higher reported weekly cycling. Lastly, the proportion who consumed 1–500 g of coffee per day tended to be higher with higher reported cycling.

**Table 1:** Baseline characteristics of sample according to level of total cycling.

	Minutes of total cycling per week					
	0	1–60	61–150	151–300	>300	Total
**Participants, n**	16,589	11,711	9,424	8,756	6,033	52,513
**Sex, % women**	49.3	49.9	57.2	58.7	55.3	53.1
**Age, yrs**	56 (52–60)	55 (52–59)	56 (52–60)	55 (52–59)	55 (52–59)	56 (52–60)
**Basic school, yrs (≤7/8–10/>10)**	34.5/45.3/20.2	27.9/48.4/23.7	30.9/46.9/22.2	32.2/47.0/20.8	35.9/44.1/20.0	32.2/46.4/21.4
**Higher education, yrs (0/1–2/3–4/>4)**	16.4/22.1/39.4/22.1	11.6/20.7/42.8/24.8	13.7/22.6/41.7/22.0	14.7/25.2/39.6/20.4	16.0/25.1/37.9/21.0	14.5/22.8/40.5/22.3
**Waist circumference, cm**	90 (80–99)	89 (79–97)	87 (78–96)	86 (78–95)	86 (78–95)	88 (79–97)
LTPA other than total cycling, MET*hrs/week	42.0 (26.5–65.5)	41.0 (27.3–61.3)	45.0 (30.8–67.0)	48.0 (32.5–71.0)	57.0 (36.0–86.5)	45.0 (29.3–68.0)
**PA at work (no work/sedentary/standing/manual work/heavy manual work)**	22.1/38.1/16.4/17.9/5.4	17.8/41.5/17.5/19.1/4.1	22.8/35.1/18.3/20.1/3.7	21.5/32.8/18.1/23.6/4.1	21.9/29.0/17.8/25.7/5.7	21.1/36.4/17.4/20.4/4.6
**Smoking status/amount (never/>25 g)†**	32.2/9.8	36.5/6.0	38.0/4.6	38.6/5.0	37.5/5.5	35.9/6.7
**Alcohol intake, grams**	13.3 (5.7–32.1)	14.1 (6.9–31.5)	12.9 (6.3–28.9)	12.2 (5.6–27.0)	11.9 (4.8–30.7)	13.0 (6.0–31.0)
**Dietary energy intake, kJ**	9,327.1 (7,670.2–11,210.1)	9,456.5 (7,915.2–11,262.2)	9,499.1 (7,879.2–11,299.2)	9,596.0 (7,946.2–11,480.2)	9,958.7 (8,192.7–12,069.1)	9,505.2 (7,861.6–11,380.3)
**Polyunsaturated: saturated fat ratio**	0.4 (0.3–0.5)	0.4 (0.3–0.5)	0.4 (0.4–0.5)	0.4 (0.3–0.5)	0.4 (0.3–0.5)	0.4 (0.3–0.5)
**Vegetables, grams**	145.5 (92.8–211.1)	162.8 (108.4–226.9)	168.3 (111.9–237.3)	174.0 (115.8–247.1)	175.8 (113.9–254.8)	161.7 (105.1–230.7)
**Fruit, grams**	152.4 (78.2–259.0)	166.8 (94.8–269.9)	183.1 (105.1–291.0)	189.3 (113.1–305.0)	195.8 (112.6–323.7)	172.3 (95.3–282.0)
**Potatoes, grams**	130.7 (84.1–193.2)	128.0 (85.1–178.4)	127.0 (83.0–178.0)	128.2 (82.4–183.5)	130.3 (82.7–192.6)	128.9 (83.8–185.2)
**Wholegrain cereals, grams**	119.0 (78.3–168.1)	127.8 (88.0–173.7)	131.2 (90.2–177.3)	135.8 (95.0–184.3)	142.0 (99.9–196.0)	127.8 (86.6–174.9)
**Refined cereals, grams**	46.5 (29.4–77.5)	46.6 30.3–73.0)	44.7 (29.1–68.3)	45.2 (29.2–68.7)	45.7 (29.4–71.1)	45.9 (29.5–72.1)
**Glycaemic load of all carbohydrates**	173.8 (138.2–214.1)	178.4 (145.5–217.6	181.2 (146.6–221.4)	185.3 (149.3–226.8)	191.8 (154.9–236.4)	180.1 (144.6–220.9)
Coffee*, grams (0/1–500/501–000/>1000)	3.5/38.2/27.0/ 31.3	3.8/39.9/28.9/ 27.5	3.6/41.9/30.0/ 24.5	3.7/41.9/28.5/ 25.9	4.3/42.4/26.9/ 26.4	3.7/40.3/28.2/ 27.8

Baseline characteristics of participants according to level of total cycling. Due to skewness in distribution of continuous variables, these are presented as medians with interquartile ranges. Categorized variables are presented as proportions. Unless otherwise stated, values in grams are expressed as daily intakes.

*Categorized as above for descriptive purposes only.

†Only proportions at the two extremes are presented. yrs = years; PA = physical activity; LTPA = leisure time physical activity; MET = metabolic equivalents; hrs = hours.

Risk of T2D associated with level of total cycling is presented in Table 2. In the multivariable adjusted model, HRs (95% CI) were 1, 0.87 (0.82,0.93), 0.83 (0.77,0.89), 0.80 (0.74,0.86), and 0.80 (0.74,0.87) (*p* for trend = <0.001) for cycling 0, 1–60, 61–150, 151–300, and >300 min/wk, respectively. A significant trend indicated inverse linear relations. Additional adjustment for baseline waist circumference attenuated the associations, and HRs (95% CI) were 1, 0.97 (0.91,1.04), 0.93 (0.86,1.00), 0.92 (0.85,0.99), and 0.90 (0.82,0.98) (*p* for trend = 0.005) for cycling 0, 1–60, 61–150, 151–300, and >300 min/wk, respectively, although significance remained in all categories but 1–60 min/wk. Adjustment for baseline BMI also attenuated the associations, and HRs (95% CI) were 1, 0.96 (0.90,1.02), 0.90 (0.84,0.97), 0.89 (0.83,0.96), and 0.85 (0.78,0.93) (*p* for trend = <0.001) for cycling 0, 1–60, 61–150, 151–300 and >300 min/wk, although conserving significance in all categories except for the 1–60 min/wk category.

**Table 2:** Associated risk of T2D according to total cycling and seasonal cycling.

Total cycling(*n* = 52,513)	Cycling (median)	Cases	Person-years (PY)	Incidence rate (per 1,000 PY)	Hazard ratio: Age adjustment	Hazard ratio: Multivariable adjustment
**0**	0	2,510	228,886.2	11.0 (10.5,11.4)	1	1
**1–60**	60	1,463	166,087.8	8.8 (8.4, 9.3)	0.81 (0.76,0.86)	0.87 (0.82,0.93)
**61–150**	120	1,109	134,806.5	8.2 (7.8,8.7)	0.74 (0.69,0.80)	0.83 (0.77,0.89)
**151–300**	240	994	126,559.5	7.9 (7.4,8.4)	0.71 (0.66,0.77)	0.80 (0.74,0.86)
**>300**	450	703	86,905.3	8.1 (7.5,8.7)	0.73 (0.67,0.80)	0.80 (0.74,0.87)
***p* for trend**					<0.001	<0.001
**Seasonal cycling (*n* = 52,513)**						
**No cycling**	0	2,510	228,886.2	11.0 (10.5,11.4)	1	1
**Summer or winter**	60	1,678	191,705.8	8.8 (8.3,9.2)	0.80 (0.75,0.85)	0.88 (0.83,0.94)
**Summer and winter**	180	2,591	322,653.3	8.0 (7.7,8.3)	0.73 (0.69,0.77)	0.80 (0.76,0.85)

Analyses of total cycling and seasonal cycling on T2D risk. Cycling is presented in median minutes per week based on the average of summer and winter total cycling. The multivariable models include adjustment for age at baseline (years), sex (male/female), years of basic school (≤7/8–10/>10), years of higher education (0/1–2/3–4/>4), alcohol intake (quintiles), dietary energy intake (quintiles), vegetable intake (quintiles), fruit intake (quintiles), potato consumption (quintiles), wholegrain cereal consumption (quintiles), refined cereal consumption (quintiles), glycaemic load of all carbohydrates (quintiles), coffee consumption (quartiles), polyunsaturated:saturated fat ratio (quintiles), physical activity at work (no work/sedentary/standing/manual work/heavy manual work), smoking status (never/former/<15 g per day/15–25 g per day/>25 g per day) and leisure-time physical activity other than total cycling (quintiles). Incidence rates and hazard ratios are presented with 95% confidence intervals.

The association of seasonal cycling and T2D was also analysed (Table 2). Multivariable adjusted HRs (95% CI) were 1, 0.88 (0.83,0.94), and 0.80 (0.76,0.85) for “no cycling,” “summer or winter,” and “summer and winter,” respectively. Cycling “summer and winter” was associated with a significant lower risk of T2D compared to cycling “summer or winter.” When baseline waist circumference was added to the model, the associations were somewhat attenuated; HRs (95% CI) were 1, 0.97 (0.91,1.04), and 0.91 (0.86,0.97) for “no cycling,” “summer or winter,” and “summer and winter,” respectively, and significance remained for “summer and winter” only. Adjustment for baseline BMI also revealed an attenuation, and HRs (95% CI) were 1, 0.96 (0.90,1.02), and 0.88 (0.83,0.93) for “no cycling,” “summer or winter,” and “summer and winter,” respectively, where significance was conserved for “summer and winter” only. Notably, participants who cycled all year were exposed to a threefold higher amount of cycling compared to seasonal cyclists (180 min/wk compared to 60 min/wk). It was investigated if the estimates changed when amount of cycling during summer and/or winter was adjusted for; the estimated HRs (95% CI) were 1, 0.88 (0.83,0.94), and 0.81 (0.76,0.87) for “no cycling,” “summer or winter,” and “summer and winter,” respectively. Thus, the associations were almost unchanged, and all significance was conserved. Furthermore, cycling “summer and winter” continued to be associated with a significantly lower risk compared to cycling “summer or winter.”

In a further attempt to limit residual confounding of sports participation in the associations between cycling and T2D, separate analyses of total cycling and seasonal cycling were performed in which only those who reported no sports participation were included. Multivariable adjusted HRs (95% CI) in analysis of total cycling when only those who reported no sports participation were included (*n* = 23,765) were 1, 0.86 (0.78,0.94), 0.85 (0.77,0.94), 0.83 (0.75,0.92), and 0.85 (0.76,0.96) (*p* for trend = 0.001) for cycling 0, 1–60, 61–150, 151–300, and >300 min/wk, respectively. Similarly, for seasonal cycling (*n* = 23,765), multivariable adjusted HRs (95% CI) were 1, 0.86 (0.79,0.94), and 0.84 (0.78,0.90) for “no cycling,” “summer or winter,” and “summer and winter,” respectively. Thus, in these models, associations indicating dose response relation were less evident, although for all exposure levels of cycling, HRs were significantly lower than no cycling.

The association between changes in total cycling from baseline to the second examination and T2D risk was then investigated (Table 3). HRs (95% CI) in the multivariable adjusted model were 1, 0.88 (0.78,1.01), 0.80 (0.69,0.91), and 0.71 (0.65,0.77) for “no cycling,” “cessation,” “initiation,” and “continuation,” respectively. “Continuation” was associated with a significantly lower risk compared to both “no cycling” and “cessation”. Despite lower cumulative averages of total cycling, those who initiated cycling appeared to have a lower risk compared to those who ceased to cycle, although the difference was not statistically significant. When including waist circumference at the second examination in the model, HRs (95% CI) were 1, 0.91 (0.80,1.03), 0.85 (0.74,0.98), and 0.78 (0.71,0.86) for “no cycling,” “cessation,” “initiation,” and “continuation,” respectively, and, thus, the associations were all moderately attenuated, although no significance was lost. When adjustment for BMI at the second examination was performed, the associations were attenuated to a similar extent, and HRs (95% CI) were 1, 0.89 (0.78,1.01), 0.87 (0.75,0.99), and 0.77 (0.70,0.84) for “no cycling,” “cessation,” “initiation,” and “continuation,” respectively, but all significance remained.

**Table 3:** Associated risk of T2D according to changes in total cycling from baseline to the second examination.

(*n* = 27,712)	Cycling (median)	Cases	Person-years (PY)	Incidence rate (per 1,000 PY)	Hazard ratio: Age adjustment	Hazard ratio: Multivariable adjustment
**No cycling**	0	735	51,889.3	14.2 (13.2,15.2)	1	1
**Cessation**	30.0	335	27,247.2	12.3 (11.0,13.7)	0.87 (0.77,0.99)	0.88 (0.78,1.01)
**Initiation**	18.8	284	26,060.6	10.9 (9.7,12.2)	0.78 (0.68,0.90)	0.80 (0.69,0.91)
**Continuation**	137.5	1,469	160,159.4	9.2 (8.7,9.7)	0.65 (0.60,0.71)	0.71 (0.65,0.77)

Analysis of changes in total cycling from baseline to the second examination on T2D risk. Cycling is presented in median minutes per week of total cycling based on the cumulative average of total cycling at baseline and total cycling at the second examination. The multivariable model includes adjustment for age at the second examination (years), sex (male/female), years of basic school (≤7/8–10/>10), years of higher education (0/1–2/3–4/>4), alcohol intake (quintiles), dietary energy intake (quintiles), coffee consumption (quartiles), polyunsaturated:saturated fat ratio (quintiles), physical activity at work (no work/sedentary/ standing/manual work/heavy manual work), smoking status (never/former/<15 g per day/15–25 g per day/>25 g per day), paternal diabetes (yes/no/do not know), maternal diabetes (yes/no/do not know), and leisure-time physical activity other than total cycling (quintiles). Incidence rates and hazard ratios are presented with 95% confidence intervals.

Of those eligible for the analysis of changes in total cycling from baseline to the second examination and risk of T2D, 11,458 were excluded due to being missing in either covariates or exposure variables. Those excluded were compared to those included in regards to age, cycling exposure, waist circumference, and T2D hazard. Adjusted for sex, those excluded were marginally, although statistically, older than those included. Adjusted for age and sex, there were no differences in cycling exposure, waist circumference, and T2D hazard between included and excluded participants.

Associated risk of T2D according to level of commuter cycling is shown in Table 4. Multivariable adjusted HRs (95% CI) were 1, 0.72 (0.60,0.87), 0.83 (0.69,1.00), and 0.70 (0.57,0.85) (*p* for trend = <0.001) for 0, 1–60, 61–150, and >150 min/wk of commuter cycling, respectively. When waist circumference at the second examination was added to the model, the associations were attenuated; HRs (95% CI) were 1, 0.77 (0.64,0.92), 0.92 (0.76,1.11), and 0.78 (0.64,0.96) (*p* for trend = 0.023) for 0, 1–60, 61–150, and >150 min/wk of commuter cycling. Adjustment for BMI at the second examination attenuated the strength of the associations as well, and HRs (95% CI) were 1, 0.73 (0.61,0.88), 0.91 (0.76,1.10), and 0.79 (0.64,0.96) (*p* for trend = 0.023) for 0, 1–60, 61–150, and >150 min/wk of commuter cycling.

**Table 4:** Associated risk of T2D according to level of commuter cycling.

(*n* = 15,063)	Cycling (median)	Cases	Person-years (PY)	Incidence rate (per 1,000 PY)	Hazard ratio: Age adjustment	Hazard ratio: Multivariable adjustment
**0**	0.0	960	93,693.6	10.2 (9.6,10.9)	1	1
**1**–**60**	27.5	131	20,019.5	6.5 (5.5,7.8)	0.65 (0.54,0.77)	0.72 (0.60,0.87)
**61**–**150**	105.0	131	17,301.1	7.6 (6.4,9.0)	0.75 (0.62,0.90)	0.83 (0.69,1.00)
**>150**	229.1	105	15,826.3	6.6 (5.5,8.0)	0.65 (0.53,0.80)	0.70 (0.57,0.85)
***p* for trend**					<0.001	<0.001

Analysis of commuter cycling on T2D risk. Cycling is presented in median minutes per week of commuter cycling. The multivariable model includes adjustment for age at the second examination (years), sex (male/female), years of basic school (≤7/8–10/>10), years of higher education (0/1–2/3–4/>4), alcohol intake (quintiles), dietary energy intake (quintiles), coffee consumption (quartiles), polyunsaturated:saturated fat ratio (quintiles), physical activity at work (no work/sedentary/standing/manual work/heavy manual work), smoking status (never/former/<15 g per day/15–25 g per day/>25 g per day), paternal diabetes (yes/no/do not know), maternal diabetes (yes/no/do not know), and leisure-time physical activity other than commuter cycling (quintiles). Incidence rates and hazard ratios are presented with 95% confidence intervals.

Of those eligible for analysis of commuter cycling and risk of T2D, 5,811 were excluded due to being missing in either covariates or exposure variables. As with the previous analysis, we compared those excluded with those included in terms of age, cycling exposure, waist circumference, and T2D hazard. Adjusted for sex, those excluded were marginally, but statistically, older compared to those included. Those who were excluded reported significantly higher amount of cycling to work, but were not different in waist circumference and did not differ in T2D hazard compared to those who were included when adjusted for age and sex.

## Discussion

In this large population-based cohort study among Danish men and women residing in cycling-friendly cities, recreational and commuter cycling was associated with lower risk of T2D, and mostly in a dose-response manner, according to weekly time spent on cycling and seasonal engagement in cycling. The results of our study also suggest that initiation or continuation of cycling habits from baseline to the second examination was associated with significant T2D risk reduction compared to no cycling in the same time period. Our investigation suggested that adiposity partly mediated the associations between cycling on T2D risk.

This study expands the large body of evidence showing that physical activity is associated with a lower risk of T2D [8] by showing an association for cycling specifically. The finding that increased total cycling was associated with lowered T2D risk adds to evidence from prospective studies that have compared cyclists and non-cyclists free of chronic disease, which have found no relationship [13,14], and expands upon evidence from cross-sectional studies that have reported lower prevalence of T2D associated with total cycling engagement [9,10]. The positive associations between commuter cycling and T2D builds upon evidence from a Finnish prospective study, which found positive associations between commuter walking and cycling pooled and T2D [32], and cross-sectional studies that have found a positive association between commuter cycling and risk of diabetes [11,12]. Specifically, in a sample representative of the United Kingdom, odds ratio of diabetes (95% CI) when comparing commuter cyclists and those who reported private transport was 0.50 (0.27, 0.93) [11]. Also, among participants in the Indian Migration Study, it was shown that commuter cycling for 0, 1–29, and >30 min/d was associated with 1, 0.66 (0.44,0.98), and 0.48 (0.23,0.96) lower risk of diabetes, respectively [12]. Although not systematic, our extensive literature search in PubMed (MEDLINE) and Embase for this study found no other studies that investigated the association between seasonal cycling, as well as changes in cycling over time, on T2D risk. Those who cycled all year consistently had a lower risk of T2D compared to non-cyclists and seasonal cyclists, plausibly explained simply by the observed higher levels of cycling among those cycling all year. However, the associations did not change substantially after adjustment for amount of cycling during summer and/or winter, which indicates a benefit of cycling consistently throughout summer and winter, compared to cycling in either season only.

In the analyses of changes in total cycling over time, we found that those who took up cycling later in life had a 20% lower risk of T2D compared to those who consistently were non-cyclists. This is especially interesting due to the relatively old age of the participants at baseline. If people in late adulthood could be encouraged to start cycling habitually, the effects in terms of decreased risk may have enormous public health implications, e.g., by lowering health care expenses from treatment and care. In light of the global growing elderly population [33], such encouragement should be a central component in current and future health promotion initiatives for this population group. We also found that cycling consistently was associated with the largest decrease in risk and that ceasing cycling was associated with no decreased risk. Initiatives should thus also encourage consistent cycling throughout late adulthood and into elderly life, as well as aim to prevent cyclists from giving up cycling when approaching elderly age.

Several biological mechanisms may explain the lower risk of T2D from engaging in cycling. A trial of obese women who commuted to work found that cycling reduced fasting insulin levels after 6 mo [34]. An outdoor bicycle-based intervention in young men cycling 45 min/d for 6 wk found improvements in glucose tolerance, insulin resistance, and insulin secretion, and a tendency for decreases in fasting insulin (*p* = 0.07) [35]. In addition, exercise interventions in adults with cycle ergometers have found improvements in glucose metabolism [36–40], including decreased fasting insulin [36,38], increased insulin sensitivity [36,39], and decreased c-peptide levels [37]. However, such controlled cycling conditions may not perfectly reflect cycling in natural settings. Furthermore, a cross-sectional study found lower odds of glucose intolerance with higher reported cycling [41]. Therefore, based on findings from interventions that include either free-living or stationary cycling, as well as the results from a cross-sectional study, cycling may prevent the development of T2D by improving glucose metabolism and maintaining blood glucose within normal ranges.

A lower risk of T2D may also be explained by decreases in adiposity as a result of habitual cycling. This is supported by lower risk of overweightness and obesity among commuter cyclists observed in cross-sectional studies [11,12] and from findings in randomized controlled trials of isolated aerobic exercise in which modest reductions in weight and waist circumference were induced [42]. In the current study, when we included measures of adiposity in the regression models, the associations were strongly and consistently attenuated, suggesting a mediating role of adiposity in the relation between cycling and T2D. However, some significance was conserved even after adjustment for waist circumference or BMI, indicating that some of the associations were independent of adiposity. It is also possible that overweightness causes cessation of cycling, since previous studies suggest that adiposity also determines habitual physical activity [43], and, as a consequence, cycling exposure level may reflect previous development of adiposity. Because the measures of adiposity and cycling engagement were obtained at the same time point in our study, it is difficult to further explain the directionality of the relationships. Lastly, it is not clear whether adiposity is a confounder or a mediator in the relationship between cycling and T2D.

Several observations in our study support that the current associations are causal: there is a temporal arrangement, i.e., cycling precedes documentation of T2D; there are observed biological mechanisms that may explain the associations; the associations show mostly dose-response relationships; the lower risk is not maintained when cycling is ceased, i.e., cessation of cycling is associated with no decreased risk compared to continuation of cycling; and, lastly, similar findings have been reported previously. More research is needed, however, before a claim about causality is fully supported.

The current findings have substantial clinical relevance. Those who find difficulty in walking or jogging, e.g., obese subjects who are at increased risk of musculoskeletal complications [44], may experience health benefits from cycling, a non-weight bearing activity, despite their limitations.

In pragmatic intervention studies, summarised in a systematic review from 2010, it has previously been investigated how to promote cycling. Some studies have intervened by improving infrastructure, and others have focused on community-wide promotional activities, and modest increases in cycling have been found. Consistent positive findings have been found in studies that use individualised marketing to promote cycling. However, from a methodological standpoint, additional and more robust studies are warranted to more clearly establish how to effectively promote cycling [45]. The positive findings from studies that aim to promote cycling, combined with results from observational and small-scale experimental studies, which have shown that cycling may be valuable for chronic disease prevention, may suggest that national and local government should prioritise resources to promote cycling, e.g., making improvements in infrastructure to make urban and rural environments more cycling-friendly. This may be especially important in countries where the built environment is less compatible with cycling as a mode of transportation and recreation.

Strengths of the current study include using data from a large cohort study with a long period of follow-up, with many documented cases of T2D gathered objectively by a validated method. Limitations include use of self-reported physical activity data and, therefore, potential systematic bias and random error in estimation of exposure levels. Also, we cannot characterise documented diabetes cases according to type, although they are interpreted as T2D, since adult-onset type 1 diabetes has been found to be low in similar populations [29,30]. There may also be some limitations in terms of generalizability; the cohort is composed of Caucasian men and women 50–65 y of age at baseline, which may limit the extent to which the findings can be generalized to other ethnicities and younger populations. A limitation in the analyses is that 11,458 and 5,811 participants were excluded due to being missing in either exposure variables or covariates, in the analysis of changes in total cycling and commuter cycling, respectively. Although those excluded were significantly, albeit marginally, older, and although those excluded from the analysis of commuter cycling and T2D risk reported more cycling to work, there were no differences in T2D hazard in either of the analyses. Concern about bias due to selection from the exclusion of participants with missing data is therefore not considered a threat to internal validity. Another limitation in the analyses was the method applied for imputation of data. Multiple imputation is a well-accepted imputation strategy, which would have allowed for more data in the analyses [46]. Lastly, residual confounding or unknown confounding cannot be ruled out, although many known confounders were controlled for, which, when included in the models, consistently attenuated strengths of the associations.

In summary, in this prospective study in a large cohort of Danish men and women, we found that recreational and commuter cycling was associated with a lower risk of T2D, and we observed mostly dose–response relationships. Also, cycling all year was associated with a lower risk of T2D compared to no cycling and seasonal cycling, even after adjustment for total amount of cycling. Furthermore, continuation of cycling, as well as initiation of cycling, was associated with a substantial lower risk of T2D compared to no cycling. Because positive associations were found consistently across different approaches to analysing cycling, this adds to the robustness of the findings. Based on the results of this study, it seems beneficial to encourage adults of middle and old to engage in commuter and recreational cycling to prevent the development of T2D in late adulthood. Future cohort studies should investigate associations between cycling and T2D risk in other population groups, and experimental studies of cycling in natural settings should be conducted to further examine the possibility of effectively implementing habitual cycling in T2D prevention.

## Supporting Information

S1 Text STROBE Statement. Checklist of items that should be included in reports of observational studies.
(DOCX)

S2 Text Study proposal. (PDF)

## Abbreviations

BMI: body mass index
HRs: hazard ratios
MET: metabolic equivalent
NDR: National Diabetes Registry
T2D: type 2 diabetes
