# Supplementary material for: Associations between Recreational and Commuter Cycling, Changes in Cycling, and Type 2 Diabetes Risk: A Cohort Study of Danish Men and Women
Source: PLoS Med. 2016 Jul 12;13(7):e1002076. doi: 10.1371/journal.pmed.1002076 (PMC4942105; doi:10.1371/journal.pmed.1002076)
Supplement: S1 Text — Checklist of items that should be included in reports of observational studies. (DOCX) [file pmed.1002076.s001.docx]

STROBE Statement—checklist of items that should be included in reports of observational studies

|  | Item No. | Recommendation | Paragraph numbers per section | Relevant text from manuscript |
| --- | --- | --- | --- | --- |
| **Title and abstract** | 1 | (*a*) Indicate the study’s design with a commonly used term in the title or the abstract | Title | *“Associations between Recreational- and Commuter Cycling, Changes in Cycling, and Type 2 Diabetes risk: A Cohort Study of Danish Men and Women”* |
|  |  | (*b*) Provide in the abstract an informative and balanced summary of what was done and what was found | Abstract | *“At baseline from 1993-1997,* *24,623 men and 27,890 women from Denmark, 50-65 years of age and free of T2D and other chronic diseases, underwent a number of assessments, including completing a lifestyle questionnaire also addressing cycling habits. Approximately five years later at a second examination participants completed a new, updated lifestyle questionnaire. Cox regression was used to estimate hazard ratios of incident T2D registered in the Danish National Diabetes Registry, according to recreational- and commuter cycling and changes in cycling habits, with adjustment for a priori known T2D risk factors.. Commuter and recreational cycling was consistently associated with lower risk of T2D in Danish adults. Our results also provide evidence that late in life initiation of or continued engagement in cycling lowers risk of T2D.”* |
| Introduction | | | |  |
| Background/rationale | 2 | Explain the scientific background and rationale for the investigation being reported | 1^st^ and 2^nd^ paragraph of the Introduction section | *“Although habitual cycling is likely to be health- and fitness enhancing physical activity [4] its importance for chronic disease prevention in populations has been scarcely investigated. Habitual cycling has in prospective cohort studies of adults been associated with a lower incidence of fatal and non-fatal cardiovascular disease [5], less weight gain [6] and lower all-cause mortality [7]. Cycling may be particularly valuable for type 2 diabetes (T2D) prevention since a large body of evidence from observational and experimental studies shows that regular engagement in physical activity and lifestyle intervention incorporating physical activity substantially decreases T2D risk [8].*  *The benefits of recreational and commuter cycling for the prevention of T2D are still unclear; cycling has been association with a lower risk of diabetes cross-sectionally [9-12] and two prospective studies found no lower risk of incident T2D when comparing cyclists with non-cyclists who were free of chronic disease at recruitment [13, 14]. It remains to be examined in greater detail whether cycling is valuable in the prevention of T2D.”* |
| Objectives | 3 | State specific objectives, including any prespecified hypotheses | 2^nd^ paragraph of the Introduction section | *“The purpose of this study was to investigate the prospective association of recreational- and commuter cycling with T2D risk among generally healthy Danish adults in the Diet, Cancer and Health cohort study. We also investigated how seasonal cycling and changes in cycling habits were related to incidence of T2D.”* |
| Methods | | | |  |
| Study design | 4 | Present key elements of study design early in the paper | 2^nd^ paragraph of the Introduction | *“The purpose of this study was to investigate the prospective association of recreational- and commuter cycling with T2D risk among generally healthy Danish adults in the Diet, Cancer and Health cohort study.”* |
| Setting | 5 | Describe the setting, locations, and relevant dates, including periods of recruitment, exposure, follow-up, and data collection | 1^st^ and 2^nd^ paragraph of the Participants section | *“Between 1993 and 1997, 80,996 men and 79,729 women were invited to participate in the Diet, Cancer and Health study. Inhabitants of Aarhus and Copenhagen and surrounding cities were invited if between 50 and 64 years of age, born in Denmark and without a diagnosis of cancer registered in the Danish Cancer Registry .. Approximately five years later (mean 5.4 years) between 1999 and 2003 participants still alive and residing in Denmark were invited for a second examination”* |
| Participants | 6 | (*a*) ***Cohort study***—Give the eligibility criteria, and the sources and methods of selection of participants. Describe methods of follow-up  *Case-control study*—Give the eligibility criteria, and the sources and methods of case ascertainment and control selection. Give the rationale for the choice of cases and controls  *Cross-sectional study*—Give the eligibility criteria, and the sources and methods of selection of participants | 1^st^ and 2^nd^ paragraph of the Participants section | *“Inhabitants of Aarhus and Copenhagen and surrounding cities were invited if between 50 and 64 years of age, born in Denmark and without a diagnosis of cancer registered in the Danish Cancer Registry .. Approximately five years later (mean 5.4 years) between 1999 and 2003 participants still alive and residing in Denmark were invited for a second examination and 45,264 or 79.3% of the original cohort participated.”* |
|  |  | (*b*) ***Cohort study***—For matched studies, give matching criteria and number of exposed and unexposed  *Case-control study*—For matched studies, give matching criteria and the number of controls per case |  | Not applicable |
| Variables | 7 | Clearly define all outcomes, exposures, predictors, potential confounders, and effect modifiers. Give diagnostic criteria, if applicable | - 1^st^ paragraph of the Follow-up and identification of T2D cases section (outcome)  - 1^st^-5^th^  paragraph of the Assessment of physical activity section  (exposure)  - 6^th^ paragraph of the Statistical methods section (confounders) | *“Cases of diabetes were identified based on registration in the Danish NDR. Established in 2006, NDR identifies cases based on data from other Danish medical registries. If one of six specific criteria in one of three registries is met, this person is included. These criteria are; a diagnosis of diabetes according to the National Patient Registry; registration of diabetic chiropody, i.e. treatment of feet when also registered as a diabetic patient, in the National Health Service Register; five annual blood glucose measurements, or two blood glucose measurements per year for five consecutive years, according to the National Health Service Register; and, lastly purchase of anti-diabetic drugs or prescribed insulin, registered in the Danish National Prescription Registry”*  *“In the lifestyle questionnaires, physical activity was enquired for both summer and winter, except for physical activity at work and for stair climbing. Participants were asked to report total hours/week of physical activity, although at the second examination frequency of the activity was specified, as well as duration per time the activity was performed. This information was used to calculate total hours/week for each activity..* *Total cycling was categorized in 0, 1-60, 61-150, 151-300 and >300 minutes/week. ..* *Subjects were also categorized according to seasonal cycling as; non-cyclist, cyclists only summer or winter, or cyclist summer and winter.. Participants were then grouped according to those who did no cycling, ceased to-, initiated- or continued cycling in the period between baseline and the second examination.. Commuter cycling was categorized similarly as total cycling, although because there were few incident T2D cases in the two highest categories these were merged to >150 minutes/week.”*  *“Confounders included in the cox models were selected based on knowledge a priori. For each analysis an age-adjusted and a multivariable adjusted model was computed .. the following variables were adjusted for ..”*  We identified no effect modifiers |
| Data sources/ measurement | 8* | For each variable of interest, give sources of data and details of methods of assessment (measurement). Describe comparability of assessment methods if there is more than one group | 1^st^ and 2^nd^ paragraph of the Data collection section | *“At baseline a validated semi-quantitative food frequency questionnaire, which was developed to be compatible with the Danish diet [16-19], was sent by mail and filled out before a visit to the study clinic. At the clinic an additional questionnaire was completed, containing lifestyle questions in general, addressing habits pertaining to e.g. physical activity, smoking and alcohol. Furthermore, a lab technician measured anthropometrics, including weight, height and waist circumference, and biological material was collected [15].*  *At the second examination a similar dietary survey, which also included questions on foods that since baseline had been introduced to the Danish diet, was sent by mail and completed in the participants’ home. An updated lifestyle questionnaire was also sent to the participants, e.g. introducing questions on social network and self-rated health from the Medical Outcomes Study 36-item short-form survey. Enquiry of weight according to self-report was included in the lifestyle questionnaire. A tape measure was sent also to self-assess waist circumference”* |
| Bias | 9 | Describe any efforts to address potential sources of bias | - 6^th^ paragraph of the Statistical methods section  - 7^th^ and 9^th^ paragraph of the Results section | - “Confounders included in the  cox models were selected based  on knowledge a priori ... “  (Extensive adjustment for a  priori known confounders was  conducted to avoid bias due to confounding)  We compared those excluded because of missing data in either covariate or exposure variables with those included in the analytic sample, to investigate if selection bias at this stage was present |
| Study size | 10 | Explain how the study size was arrived at | 1^st^ and 2^nd^ paragraph of the Participants section | *Between 1993 and 1997, 80,996 men and 79,729 women were invited to participate .. Eligible persons were identified through the Civil Registration System and 27,178 men and 29,875 women agreed to participate in baseline examination [15]. In analysis of total cycling (combined recreational and commuter cycling) and seasonal cycling, which was reported at baseline, the following disease cases were excluded; diabetes according to the National Diabetes Registry (NDR), the National Patient Registry or self-report (n=1,395); also acute myocardial infarction (n=913); stroke (n=597); and cancer (n=569). This corresponded to exclusion of 3,268 participants registered with one or more chronic diseases, leaving 53,785 eligible participants. After excluding missing data in covariates and exposure variables 52,513 participants were included in the analyses.*  *Approximately five years later (mean 5.4 years) between 1999 and 2003 participants still alive and residing in Denmark were invited for a second examination and 45,264 or 79.3% of the original cohort participated. In analysis of changes in total cycling from baseline to the second examination, new disease cases between the two examinations were excluded, in addition to those listed above; diabetes according to the NDR, the National Patient Registry or self-report (n=1,485); also acute myocardial infarction (n=463); stroke (n=368); and cancer (n=2,206). This corresponded to exclusion of 4,328 participants registered with one or more chronic diseases. When also excluding those registered with one or more chronic diseases before baseline, a total of 6,094 participants were excluded, leaving 39,170 eligible participants. When those with missing data in covariates and exposure variables were excluded, 27,712 participants were included in the analysis. For analysis of commuter cycling, which was reported at the second examination, unemployed individuals (n=1,375) and those who had retired (n=20,738) were excluded also, leaving 20,874 eligible participants. After excluding missing data in covariates and exposure variables 15,063 participants were included in the analysis (see Figure 1)”.* |

Continued on next page

| Quantitative variables | 11 | Explain how quantitative variables were handled in the analyses. If applicable, describe which groupings were chosen and why | - 3^rd^ paragraph of the Assessment of physical activity section  - 6^th^ paragraph of the Statistical Methods section | *- “Total cycling was categorized in 0, 1-60, 61-150, 151-300 and >300 minutes/week. When achieving >150 minutes/week one successfully met international recommendations for aerobic physical activity”*  *- “Dietary variables on a continuous scale were categorized into quintiles, although coffee consumption was categorized into quartiles. Leisure time physical activity variables were also categorized into quintiles. Age was treated as continuous in the model, assuming a linear relationship between age and risk of T2D.”* |
| --- | --- | --- | --- | --- |
| Statistical methods | 12 | (*a*) Describe all statistical methods, including those used to control for confounding | 4^th^ paragraph of the Statistical Methods section | *“Hazard ratios (HRs) with 95% CIs of developing T2D according to cycling habits were estimated using Cox Proportional Hazard Regression”* |
|  |  | (*b*) Describe any methods used to examine subgroups and interactions | 7^th^ paragraph of the Statistical Methods section | *“We also statistically evaluated whether there was an interaction between sex and cycling on risk of T2D in the analyses. This was done by comparing models with an interaction term and main effects with models containing main effects only, using the likelihood-ratio test.”* |
|  |  | (*c*) Explain how missing data were addressed | 2^nd^ paragraph of the Statistical Methods section | *“If participants had only answered for one season in physical activity variables, the value for the unanswered season was imputed based on the means for summer and winter for participants of the same sex and age (six strata) who had answered the question for both seasons.”* (Thus we imputed missing data in physical activity variables) |
|  |  | (*d*) ***Cohort study***—If applicable, explain how loss to follow-up was addressed  *Case-control study*—If applicable, explain how matching of cases and controls was addressed  *Cross-sectional study*—If applicable, describe analytical methods taking account of sampling strategy | 4^th^ paragraph of the Statistical Methods section | Subjects who were lost to follow up  were still included in the analyses  via censoring: *“Right truncation*  *was on the date of the following;*  *event, death, emigration, inactivity*  *or change in the Civil Registration*  *System, or end of follow-up*  *(31/12/2011), whichever came*  *first.”* |
|  |  | (*e*) Describe any sensitivity analyses | 2^nd^ paragraph of the Statistical Methods section | We compared associations from the analyses with and without imputation of physical activity variables: *“The multivariable analyses performed without imputation of physical activity variables, however showed almost identical results as the analyses with imputation, with no differences in direction of associations or statistical significance.”* |
|  | | | | |
| Participants | 13* | (a) Report numbers of individuals at each stage of study—eg numbers potentially eligible, examined for eligibility, confirmed eligible, included in the study, completing follow-up, and analysed | 1^st^ and 2^nd^ paragraph of the Participants section | *Between 1993 and 1997, 80,996 men and 79,729 women were invited to participate .. Eligible persons were identified through the Civil Registration System and 27,178 men and 29,875 women agreed to participate in baseline examination [15]. In analysis of total cycling (combined recreational and commuter cycling) and seasonal cycling, which was reported at baseline, the following disease cases were excluded; diabetes according to the National Diabetes Registry (NDR), the National Patient Registry or self-report (n=1,395); also acute myocardial infarction (n=913); stroke (n=597); and cancer (n=569). This corresponded to exclusion of 3,268 participants registered with one or more chronic diseases, leaving 53,785 eligible participants. After excluding missing data in covariates and exposure variables 52,513 participants were included in the analyses.*  *Approximately five years later (mean 5.4 years) between 1999 and 2003 participants still alive and residing in Denmark were invited for a second examination and 45,264 or 79.3% of the original cohort participated. In analysis of changes in total cycling from baseline to the second examination, new disease cases between the two examinations were excluded, in addition to those listed above; diabetes according to the NDR, the National Patient Registry or self-report (n=1,485); also acute myocardial infarction (n=463); stroke (n=368); and cancer (n=2,206). This corresponded to exclusion of 4,328 participants registered with one or more chronic diseases. When also excluding those registered with one or more chronic diseases before baseline, a total of 6,094 participants were excluded, leaving 39,170 eligible participants. When those with missing data in covariates and exposure variables were excluded, 27,712 participants were included in the analysis. For analysis of commuter cycling, which was reported at the second examination, unemployed individuals (n=1,375) and those who had retired (n=20,738) were excluded also, leaving 20,874 eligible participants. After excluding missing data in covariates and exposure variables 15,063 participants were included in the analysis (see Figure 1)”.* |
|  |  | (b) Give reasons for non-participation at each stage |  | See immediately above |
|  |  | (c) Consider use of a flow diagram |  |  |
| Descriptive data | 14* | (a) Give characteristics of study participants (eg demographic, clinical, social) and information on exposures and potential confounders | 2^nd^ paragraph of the Results section | *“At baseline cyclists differed from non-cyclists on most characteristics (Table 1). With higher weekly time spent cycling median waist circumference was lower. In relation to physical..”* |
|  |  | (b) Indicate number of participants with missing data for each variable of interest | 1^st^ and 2^nd^ paragraph of the Participants section | See Methods section in the  description of “Participants” (for  each analysis) when those eligible  for the study are listed following  those who are actually included in the analyses:  *“ultimately leaving .. eligible*  *participants. After excluding*  *missing data in covariates and*  *exposure variables .. participants*  *were included in the analyses.”* |
|  |  | (c) *Cohort study*—Summarise follow-up time (eg, average and total amount) | 1^st^ paragraph of the Results section | *“The current study included 743,245.4 person-years of follow-up (mean follow-up 14.2 years) from 52,513 participants in which 6,779 incident cases of T2D were documented. This corresponded to an incidence rate of 9.1 (8.9,9.3) cases per 1000 years of follow-up.”* |
| Outcome data | 15* | *Cohort study*—Report numbers of outcome events or summary measures over time |  | See immediately above. Also in table 2-4 the amount of outcome events is summarized by exposure categories. |
|  |  | *Case-control study—*Report numbers in each exposure category, or summary measures of exposure |  |  |
|  |  | *Cross-sectional study—*Report numbers of outcome events or summary measures |  |  |
| Main results | 16 | (*a*) Give unadjusted estimates and, if applicable, confounder-adjusted estimates and their precision (eg, 95% confidence interval). Make clear which confounders were adjusted for and why they were included | 4^th^ and 6^th^ paragraph of the Statistical Methods section | *“Hazard ratios (HRs) with 95% CIs of developing T2D according to cycling habits were estimated using Cox Proportional Hazard Regression… Confounders included in the cox models were selected based on knowledge a priori. For each analysis an age-adjusted and a multivariable adjusted model was computed .. the following variables were adjusted for;”* |
|  |  | (*b*) Report category boundaries when continuous variables were categorized | 3^rd^ paragraph of the Results section | *“In the multivariable adjusted model HRs were 1, 0.87 (0.82,0.93), 0.83 (0.77,0.89), 0.80 (0.74,0.86) and 0.80 (0.74,0.87) (p for trend = <0.001) for cycling 0, 1-60, 61-150, 151-300 and >300 minutes/week, respectively ..”* |
|  |  | (*c*) If relevant, consider translating estimates of relative risk into absolute risk for a meaningful time period |  |  |

Continued on next page

| Other analyses | 17 | Report other analyses done—eg analyses of subgroups and interactions, and sensitivity analyses | - 5^th^ paragraph of the Results section  (Subgroup analyses)  - 7^th^ paragraph of the Statistical Methods section  (Interaction)  - 2^nd^ paragraph of the Statistical Methods section (sensitivity analysis) | *“In further attempt to limit residual confounding of sports participation in the associations between cycling and T2D, separate analyses of total cycling and seasonal cycling were performed where only those who reported no sports participation were included.”*  *“We also statistically evaluated whether there was an interaction between sex and cycling on risk of T2D in the analyses. This was done by comparing models with an interaction term and main effects with models containing main effects only, using the likelihood-ratio test. No interaction of sex was found in any analyses.”*  We compared associations from the analyses with and without imputation of physical activity variables: *“The multivariable analyses performed without imputation of physical activity variables, however showed almost identical results as the analyses with imputation, with no differences in direction of associations or statistical significance.”* |
| --- | --- | --- | --- | --- |
| Discussion | | | | |
| Key results | 18 | Summarise key results with reference to study objectives | 1^st^ paragraph of the Discussion section | *“In this large population-based cohort study among Danish men and women residing in cycling friendly cities, recreational and commuter cycling was associated with lower risk of T2D and mostly in a dose-response manner according to weekly time spent on cycling and seasonal engagement in cycling. The results of our study also suggest that initiation or continuation of cycling habits from baseline to the second examination was associated with significant T2D risk reduction compared to no cycling in the same time period. Our investigation suggested that adiposity partly mediated the associations between cycling on T2D risk.”* |
| Limitations | 19 | Discuss limitations of the study, taking into account sources of potential bias or imprecision. Discuss both direction and magnitude of any potential bias | 9^th^ paragraph of the Discussion section | *“Limitations include use of self-reported physical activity data and therefore potential systematic bias and random error in estimation of exposure levels. Also we cannot characterize documented diabetes cases according to type, although they are interpreted as mainly T2D, as adult-onset type 1 diabetes has been found to be low in similar populations [28, 29]. There may also be some limitations in terms of generalizability; the cohort is composed of Caucasian men and women 50-64 years of age at baseline, which may limit the extent to which the findings can be generalized to other ethnicities and younger populations. A limitation in the analyses is that 11,458 and 5,811 participants were excluded due to being missing in either exposure variables or covariates, in the analysis of changes in total cycling and commuter cycling, respectively. Although those excluded were significantly, albeit marginally older, and although those excluded from the analysis of commuter cycling and T2D risk reported more cycling to work, there were no differences in T2D hazard in either of the analyses. Concern about bias due to selection from the exclusion of participants with missing data is therefore not considered a threat to internal validity. Another limitation in the analyses was the method applied for imputation of data. Multiple imputation is a well-accepted imputation strategy, which would have allowed for more data in the analyses [45]. Lastly, residual confounding or unknown confounding cannot be ruled out, although many known confounders were controlled for, which, when included in the models, consistently attenuated strengths of the associations.”* |
| Interpretation | 20 | Give a cautious overall interpretation of results considering objectives, limitations, multiplicity of analyses, results from similar studies, and other relevant evidence | 10^th^ paragraph of the Discussion section | *“Based on the results of this study it seems beneficial to encourage adults of middle and old to engage in commuter and recreational cycling to prevent the development of T2D in late adulthood.”* |
| Generalisability | 21 | Discuss the generalisability (external validity) of the study results | 9^th^ paragraph of the Discussion section | *“There may also be some limitations in terms of generalizability; the cohort is composed of Caucasian men and women 50-65 years of age at baseline, which may limit the extent to which the findings can be generalized to other ethnicities and younger populations.”* |
| Other information | |  | | |
| Funding | 22 | Give the source of funding and the role of the funders for the present study and, if applicable, for the original study on which the present article is based |  | See “Financial disclosure” |

*Give information separately for cases and controls in case-control studies and, if applicable, for exposed and unexposed groups in cohort and cross-sectional studies.

**Note:** An Explanation and Elaboration article discusses each checklist item and gives methodological background and published examples of transparent reporting. The STROBE checklist is best used in conjunction with this article (freely available on the Web sites of PLoS Medicine at http://www.plosmedicine.org/, Annals of Internal Medicine at http://www.annals.org/, and Epidemiology at http://www.epidem.com/). Information on the STROBE Initiative is available at www.strobe-statement.org.
