# Supplementary material for: Associations between Recreational and Commuter Cycling, Changes in Cycling, and Type 2 Diabetes Risk: A Cohort Study of Danish Men and Women
Source: PLoS Med. 2016 Jul 12;13(7):e1002076. doi: 10.1371/journal.pmed.1002076 (PMC4942105; doi:10.1371/journal.pmed.1002076)
Supplement: S2 Text — (PDF) [file pmed.1002076.s002.pdf]

January 2013

Study proposal:
Commuting by bicycle to work and during leisure and risk of type 2 diabetes, coronary heart disease, and premature mortality in Danish men and women

Project group: Anders Grøntved, Lars Østergaard, Kim Overvad, Majken K. Jensen (flere...)

Outline
There is a great interest in disentangling and quantifying which particular forms of physical activities are associated with decreased risk of morbidities and mortality. Two important physical activities are bicycling and walking as these may be easily adopted and maintained in everyday life in commuting friendly environments such as those in the Nordic countries. Whereas walking previously has been associated with a reduced risk of type 2 diabetes, coronary heart disease and all-cause mortality independent of time spent on other physical activity in different populations around the world, very few studies have specifically assessed the health benefits associated with bicycling. This study will aim to quantify the association of commuting by bicycle to work and during leisure with the risk of type-2 diabetes, coronary heart disease, and mortality from cardiovascular diseases, and all-causes in the Diet, Cancer and Health cohort where bicycling to work and during leisure are likely to be common. Furthermore, the study will aim to estimate the population impact of bicycling by estimating the proportion of deaths and cases of type-2 diabetes and coronary heart disease that could be prevented if everyone in this cohort started bicycling to work or during leisure time.

Methods
Study population
Between December 1993 and May 1997, a total of 160,725 persons aged 50 to 64 years were invited to participate in the Danish prospective study "Diet, Cancer and Health". Eligible participants were inhabitants of the greater Copenhagen or Aarhus areas, born in Denmark, and with no records of cancer registered in the Danish Cancer Registry at the time of invitation. In all, 27,178 men (33.6% of total number eligible) and 29,875 women (37.5% of total number eligible) participated.

The study was conducted in accordance with the Helsinki Declaration II and approved by the Ethical Committees on Human Studies for the Copenhagen and the Aarhus municipalities (KF 01-116/96).

Bicycling and walking to work and during leisure
Information on bicycling and walking to work and during leisure time were obtained in two separte questions divided in the summer- and winter season. In these questions, the number of hours/week of bicycling and walking respectivley was collected. The

mean of summer and winter hours/week of bicycling and walking respectively will be calcuated.

Covariates

Questionnaire variables

Age, gender, educational level, family history of diabetes/CVD, smoking status, frequency of alchohol consumption, alcohol intake, coffee consumption, medication for dyslipidemia and hypertension, occupational phyical activity, non-exercise physical activity (house- and garden work), sports participation, and the dietary variables total energy intake, cereal fiber intake, whole grain intake, fruit and vegetable intake, glycemic load, the ratio of unsaturated to saturated fat, and intake of trans-fat derived from the 192-item food frequency questionnaire (FFQ).

Clinical examination variables

Height, weight, and waist circumference.

Registriation of end-points during follow-up

The study population will be followed by linkage with central Danish registries using the unique person identification number. Prevalent cases of cardiovascular diseases, cancer (Danish Cancer Registry), and diabetes at baseline will be excluded. Record linkage to the Central Population Registry will give information on vital status, date of death, and date of emigration.

Coronary heart disease, type 2 diabetes, and death

Cases of fatal- and non-fatal coronary heart disease and diabetes between baseline and death, emigration, or the end of follow-up (whatever occurred first) will be identified from The Danish National Registry of Patients, the National Mortality Files, or the Danish National Diabetes Registry.

All participants with missing information on variables considered in the analyses will be excluded.

Follow-up measures of exposures

At follow-up in 2003-2007 (some) participants also reported their physical activity, including commuting activity, and other covariates described above.

Analysis plan

Cox proportional hazard regression wilth age as the time scale will be used to estimate the association of bicycling and walking to work or during leisure time with the risk of type 2 diabetes, coronary heart disease, and mortality from cardiovascular diseases, and all-causes. Analyses will be corrected for delayed entry. Bicycling and walking will be modeled as categories (none, >0-1 hours/week, >1-2 hours/week, 2-3 hours/week, >3 hours/week (if the distribution of walking and bicycling allows these groups)) and as per hours/week assuming a linear relationship of the natural logarithm of RR with increasing time spent on these activities. Restricted cubic spline regression will also be

carried out to examine if the associations are non-linear.

Age-and gender adjusted analyses will be carried out and multivariable adjusted analyses additionally including the following covariates: educational level (<8, 8-10, and >10 years), family history of diabetes/CVD (yes/no), smoking status (never, former, current smokers of 1-14, 15-24 or >24 grams per day), frequency of alcohol consumption (never, <1/month, 1–3 times/month, 1 time/week, 2–4 times/week, 5–6 times/week, daily), alcohol intake (g/day), coffee consumption (quintiles), medication for dyslipidemia (yes/no) and hypertension (yes/no), occupational phyical activity (ordinal variable 1-4), non-exercise physical activity (quintiles), sports participation (quintiles), total energy intake, cereal fiber intake, whole grain intake, fruit and vegetable intake, glycemic load, the ratio of unsaturated to saturated fat, and intake of trans-fat (dietary variabels in quintiles). Biciclying and walking to work and during leisure time will be mutually adjusted.

Assuming a causal and unbiased association of bicycling with the risk of diabetes, coronary heart disease, and mortality, population attributable fractions (PAF) will be calculated to estimate the proportion of cases that might be avoided in this population if everyone in the cohort started bicycling to work or during leisure time. Confidence intervals (95%) of the estimated PAF will be calculated using the delta method. PAF will also be calculated as per hours per week of bicycling.

Heterogeneity in estimates of associations by gender, overweight status, sports participation, non-exercise physical activity, and educational level will be explored.

To further limit the possibility of confounding by other physical activity beyond bicycling, an analysis restricted to participants reporting no engagement in sports, occupational physical activity, and non-exercise activity will be conducted (assuming adequate number of participants with such characteristics).

Restricted to participants with two repeated assessments of exposure, additional analyses will also conducted analyzing change in bicycling and walking to work and during leisure. In these analysis cases of cardiovascular diseases, cancer, and diabetes at baseline and from baseline to second assessment of exposure will be excluded. Furtermore, analyses with weight gain and incident overweight/obesity at follow-up as the outcome will be carried out in a linear- and logistic regression model respectivey adjusting for baseline levels of weight or bmi and other covariates.
